# Physical inactivity prevalence and associated factors among iranian older adults in the 2021 STEPS survey

**DOI:** 10.1038/s41598-026-42828-x

**Published:** 2026-03-06

**Authors:** Arya Khezrpour, Sheida Sarrafzadeh, Mahbube Ebrahimpur, Mohanna Khojaste, Nazila Rezaei, Moloud Payab, Farshad Sharifi

**Affiliations:** 1 Non-Communicable Diseases Research Center, Endocrinology and Metabolism Population Sciences Institute, Tehran University of Medical Sciences, Tehran, Iran; 2 School Of Medicine, Tehran University of Medical Sciences, Tehran, Iran; 3 Department of Epidemiology and Biostatistics, School of Health, Tehran University of Medical Sciences, Tehran, Iran; 4 Endocrinology and Metabolism Research Center, Endocrinology and Metabolism Clinical Sciences Institute, Tehran University of Medical Sciences, Tehran, Iran; 5 Elderly Health Research Center, Endocrinology and Metabolism Population Sciences Institute, Tehran University of Medical Sciences, Jalal-Al-Ahmad Street, North Kargar Avenue, 14117 − 13137 Tehran, Iran

**Keywords:** Physical Inactivity, Sedentary Behavior, Aged, STEPS, Socioeconomic Factors, Multimorbidity, Sex Characteristics, Diseases, Health care, Medical research, Risk factors

## Abstract

Physical inactivity is a major, modifiable driver of non-communicable diseases in aging populations. We quantified the prevalence of physical inactivity among Iranian older adults and identified demographic, socioeconomic, and health-related correlates. We analyzed nationally representative 2021 STEPS data from 5,491 adults aged ≥ 60 years. Physical activity was assessed via World Health Organization Global Physical Activity Questionnaire (WHO GPAQ) and categorized as sufficient (≥ 600 MET-min/week) vs. insufficient (< 600). Sedentary time (min/day) was recorded. Complex survey design was accounted for in estimates. Associations with inactivity were examined using sex-stratified generalized linear mixed-effects models adjusting for age, education, marital and employment status, urban or rural residence, wealth tertile, obesity, depressive symptoms, and multimorbidity. Among the participants, mean sedentary time was 288 min/day and mean total activity was 1,326 MET-min/week. Overall, 69.6% (95% CI: 68.3–71.0) were inactive, higher in women (76.8%, 75.0–78.5) than men (62.4%, 60.3–64.4). Inactivity was highest in adults aged ≥ 80 years (80.9%, 77.1–84.7). Urban residence was associated with higher odds of inactivity (adjusted OR [aOR] 1.35, 95% CI: 1.13–1.61) and higher education (≥ 12 years formal education) showed lower odds (aOR 0.73, 0.61–0.88). Obesity and depressive symptoms were each related to higher odds of inactivity (aOR 1.34, 1.08–1.66 and aOR 1.30, 1.12–1.51); odds were progressively higher across multimorbidity categories (e.g., ≥ 3 conditions, aOR 1.26, 1.02–1.55 vs. none). Sex differences were evident. Compared with ages 60–69, adults aged ≥ 80 had higher odds of inactivity in women (aOR 3.20, 95% CI 1.96–5.24) than in men (aOR 1.91, 1.41–2.60). Urban residence and higher wealth were also related to higher odds in women (urban aOR 1.62, 1.25–2.11; wealth Q3 aOR 1.41, 1.14–1.75), while higher wealth was related to lower odds in men (Q3 aOR 0.76, 0.61–0.94). Physical inactivity is highly prevalent among Iranian older adults, especially women, the oldest age groups, urban residents, and those with obesity, depression, or multiple chronic conditions. Findings support gender-responsive, multicomponent strategies—including community programs, primary-care integration, and activity-supportive urban design—to reduce inactivity and advance healthy aging in Iran.

**Supplementary Information:**

The online version contains supplementary material available at 10.1038/s41598-026-42828-x.

## Introduction

Global population aging is a defining demographic shift of the 21 st century. People aged ≥ 60 years will more than double from one billion in 2020 to 2.1 billion by 2050, with most living in low- and middle-income countries (LMICs)^1^. This rapid demographic transition is accompanied by an epidemiological shift, where the burden of non-communicable diseases (NCDs)—such as cardiovascular disease (CVD), diabetes mellitus (DM), chronic respiratory conditions, and dementia—has grown substantially, placing immense strain on health systems, particularly in resource-limited settings^2^.

A key contributor to the rising prevalence of NCDs is physical inactivity. More than one third of adults do not meet the World Health Organization (WHO) recommendations for physical activity. Additionally, the prevalence of inactivity increases significantly with age^3^. Insufficient physical activity is a leading modifiable risk factor for global mortality—contributing to over five million premature deaths annually—and incurring substantial economic costs^4,5^.

The determinants of physical inactivity in older adults are complex and multifactorial. Inactivity is not distributed equally across populations; women are consistently less active than men, and disparities exist across socioeconomic strata, with lower levels of education and income being associated with higher rates of inactivity^3,6^. Behavioral and clinical factors also play a critical role. For instance, smoking, obesity, and poor diet are often correlated with lower activity levels^6^. Furthermore, psychosocial factors such as depression and weak social support can create significant barriers to staying active^7^, while the presence of chronic health conditions may paradoxically lead to greater inactivity due to pain, fatigue, or fear of exacerbating symptoms^8^.

Understanding these multi-level determinants is essential for designing effective public health interventions that promote active aging and reduce the NCD burden. This is particularly crucial in LMICs like Iran, which are concurrently managing population aging and a high prevalence of chronic disease risk factors. Utilizing data from the 2021 Iranian STEPwise approach to chronic disease risk factor surveillance (STEPS) survey, this study aims to investigate the patterns and correlates of physical activity’s factors among older adults in Iran. Specifically, we will examine the association between physical activity levels, sedentary behavior, and NCD risk factors, and identify the key demographic, socioeconomic, and health-related determinants shaping activity levels in this population. By elucidating these factors, this research seeks to inform targeted strategies to enhance active aging in Iran.

## Methods

### Study population

**Fig. 1:**
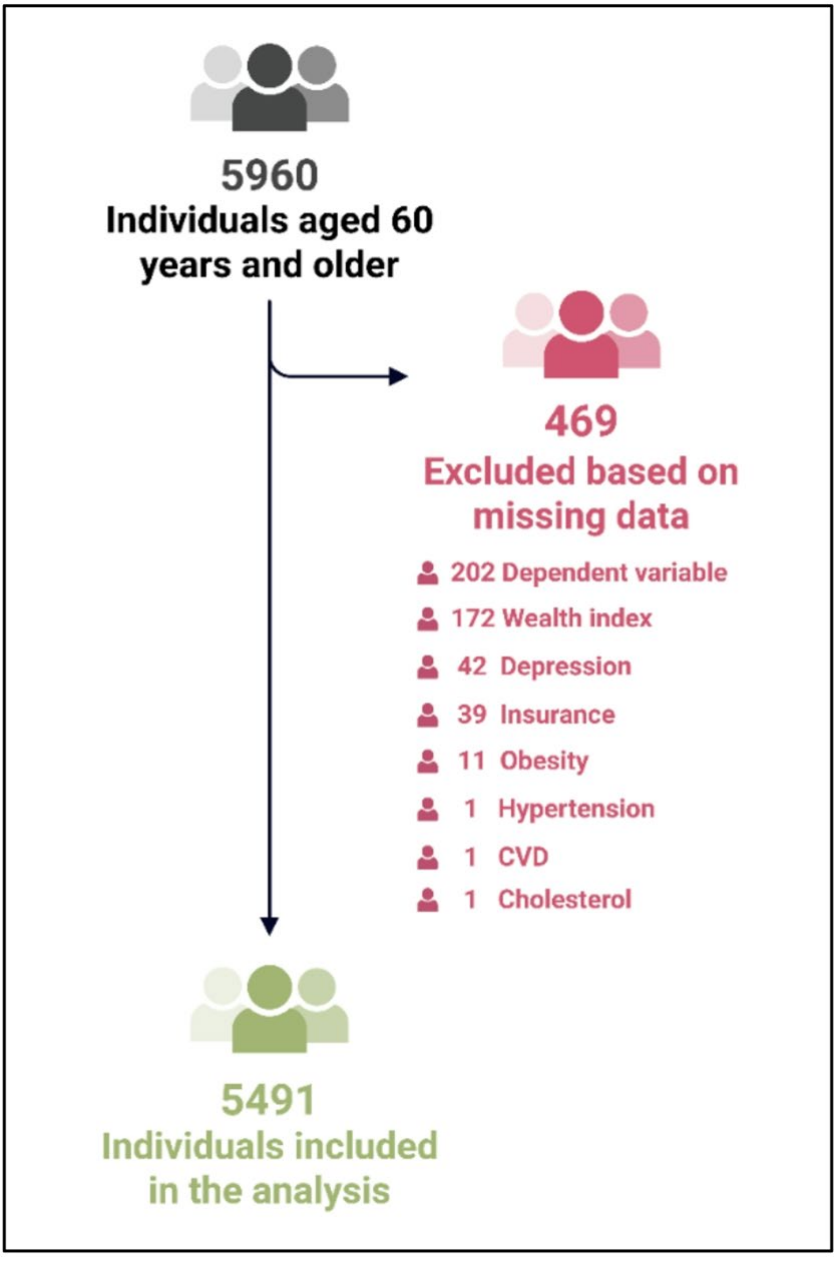
Study population and missing data flow chart.

The analysis was based on data obtained from the STEPS study dataset. People with psychological problems who may be unable to answer the questionnaire, people for whom anthropometry measurement was impossible due to physical problems and people who could not provide laboratory samples were excluded from the study. A total of 5,960 participants aged ≥ 60 years were identified. Of total, 469 (7.8%) were excluded for missing data, detailed in the flowchart (Fig. 1). Since the mechanism of missingness was completely at random (MCAR), complete deletion approach was considered. The final analytic sample comprised 5,491 older adults. Data on demographic factors and metabolic and behavioral risk factors were obtained from the included participants.

### Variables and definitions

Participants’ age was categorized into three groups (60–69, 70–79, and ≥ 80 years). Educational attainment was classified by years of formal schooling (0–6, 7–11, and ≥ 12 years). Marital status was dichotomized as married versus unmarried (never married, divorced, or widowed), and employment status as employed versus not employed (including unemployed, homemaker, or retired).

All measurements were performed by trained health technicians following standardized WHO STEPS protocols. Waist circumference (WC) was measured in centimeters using a non-stretchable measuring tape at the midpoint between the lower margin of the last palpable rib and the top of the iliac crest. WC was recorded as a single measurement per WHO STEPS procedures; therefore, an intra-participant coefficient of variation or technical error could not be estimated from the dataset, and some between-technician measurement variability may remain. Blood pressure was measured two times on the participant’s right arm after five minutes of seated rest, using a validated automated oscillometric device. The average of two readings was used for analysis.

Central obesity was defined by WC ≥ 95 cm and those with WC < 95 were considered normal. Socioeconomic status was measured using a household wealth index, constructed from asset ownership and housing characteristics and divided into tertiles, from the poorest (Q1) to the richest (Q3)^9,10^.

DM was defined as fasting plasma glucose (FPG) ≥ 126 mg/dl or self-reported use of diabetes medication^9,11^. Hypertension was defined as the mean of the last two systolic blood pressure measurements ≥ 140 mmHg, or the mean of the last two diastolic blood pressure measurements ≥ 90 mmHg, or self-reported use of antihypertensive medication^9,12^. Using the auto-analyzer (Roche-Hitachi Cobas C311, High–Technologies Corporation, Tokyo, Japan), which the reference laboratory approved, the lab test of serum total cholesterol was performed^9^ and hypercholesterolemia was defined as cholesterol > 240 mg/dl^13^. According to the manufacturer’s performance data for the Roche enzymatic total cholesterol method (CHOL2) on the Cobas platform, intermediate precision is approximately 1.4%–1.6% (coefficient of variation)^14^. Data on CVD and cancer were based on self-reports.

Multimorbidity was operationalized as the presence of any of the following five conditions: Hypertension, DM, hypercholesterolemia, CVD, or cancer diagnosed within the last 12 months. Unlike the other four conditions, which were defined irrespective of time, cancer was specifically limited to diagnosis within the past year to reflect its acute impact on physical function. These conditions were selected because they represent some of the most prevalent and impactful chronic diseases in older adults, and their association with physical inactivity is well-documented in the literature^15–19^.

Depressive symptoms were evaluated using the anxiety/depression dimension of the EuroQol five-dimensional, three-level questionnaire (EQ-5D-3 L). Participants selected one of three statements to describe their usual health state: “I am not anxious or depressed,” “I am moderately anxious or depressed,” or “I am extremely anxious or depressed”^9^. For statistical analysis, we simplified these responses into a binary indicator, classifying those who reported no problems as “having no depressive symptoms” and those who endorsed either moderate or extreme problems as “having any depressive symptoms”.

The primary outcome was physical activity, assessed using the WHO Global Physical Activity Questionnaire (GPAQ, version 2) in accordance with the STEPS protocol. Physical activity was expressed in metabolic equivalent of task (MET) minutes per week across work, transport, and recreational domains. Activities were classified as moderate- or vigorous-intensity (4 and 8 METs, respectively). Participants were categorized as sufficiently active (≥ 600 MET-min/week) or insufficiently active (< 600 MET-min/week). Sedentary behavior (minutes/day) was also recorded^9,20–24^.

### Statistical analysis

All analyses accounted for the complex survey design. Descriptive statistics included means and 95% confidence intervals (CIs) for total MET score, each activity component, and sedentary time, were computed by covariate subgroups. Prevalence of physical inactivity (total MET score less than 600 MET × min per week) with 95% CIs was similarly estimated.

To examine the association between covariates and the odds of physical inactivity while accounting for clustering, we fitted generalized linear mixed‑effects models (GLMMs) with a logit link. Given a statistically significant interaction between sex and activity levels, analyses were stratified by sex. Crude odds ratios (ORs) with 95% CIs were first estimated in univariable mixed models. Subsequently, a GLMM including all significant covariates was fitted to obtain adjusted ORs. A two‑sided type I error equal 0.05 was considered significant.

All statistical analyses were conducted in R (Version 4.5.0). Data visualization employed the ggplot2 package, and mixed‑effects modeling utilized the lme4 package.

## Results

Among the 5,491 older adults included in the study, 50.3% were women. Overall, participants averaged 288 (95% CI 282.5–293.7) min/day of sedentary time (≈ 4.8 h/day) and 1,326 (95% CI 1204–1447.2) MET-min/week of total physical activity. Women reported slightly higher sedentary time and substantially lower total MET-adjusted activity than men. Sedentary time was higher and total activity was lower in older age groups. Rural residence and current employment were associated with higher activity levels and lower sedentary time compared with urban residence and non-employment, respectively. Additional details are presented in Tables 1 and 2.

**Table 1:** Sedentary Behavior and Physical Activity Levels by Participant Characteristics.

	Variables	Subgroups	N	Sedentary (min/day)	Other (Walking) (min/week)	Leisure (Vigorous) (min/week)	Leisure (Moderate) (min/week)	Work (Vigorous) (min/week)	Work (Moderate) (min/week)	MET adjusted (MET-min/week)
		Total	5491	288.1 (282.5–293.7)	114.5 (105.8–123.2)	9.3 (6.7–11.9)	11.3 (8.8–13.8)	49.3 (39.6–59)	88.4 (77.7–99)	1325.6 (1204–1447.2)
Total	Sex	Female	2765	290.4 (282.3–298.5)	76.1 (67.6–84.5)	4.9 (2.3–7.5)	7.8 (4.8–10.8)	15.2 (10.3–20.1)	60.8 (49.6–72)	739.6 (655.9–823.2)
		Male	2726	285.8 (278–293.6)	153.5 (138.3–168.6)	13.8 (9.3–18.3)	14.9 (10.9–18.9)	83.9 (65.2–102.6)	116.3 (98.2–134.5)	1920.4 (1693.5–2147.2)
	Age (years)	60–69	3494	271.5 (265–278)	117.1 (106.6–127.5)	10.6 (7.1–14.1)	12.6 (9.3–15.9)	55.9 (42.6–69.2)	90.6 (77.4–103.8)	1412.9 (1251.5–1574.4)
		70–79	1483	308.2 (297–319.5)	122 (102.5–141.4)	6.5 (2.8–10.1)	9.9 (6–13.9)	40.3 (25.2–55.4)	95.5 (73.7–117.4)	1284.2 (1068.8–1499.5)
		≥ 80	514	346.4 (322.5–370.2)	73.9 (53.5–94.3)	8.3 (0–18.1) ^‡^	6.6 (0–15) ^‡^	29.2 (6.5–52)	51.1 (21.6–80.7)	826.7 (533–1120.4)
	Marriage	Married	4275	279.8 (273.7–286)	124.6 (114.1–135.1)	10.6 (7.5–13.7)	13.3 (10.2–16.4)	58 (46–70.1)	97.3 (84.7–109.9)	1489.9 (1340.5–1639.4)
		Not married	1216	316.9 (303.8–330.1)	79.4 (65.8–92.9)	4.7 (0.5–8.9)	4.5 (1.7–7.4)	18.9 (8.4–29.5)	57.1 (38.6–75.7)	753.1 (597.4–908.9)
	Education (years)	0–6	4027	287.5 (280.6–294.3)	107.9 (97.5–118.4)	8.1 (4.9–11.3)	9.2 (6.3–12.2)	50.1 (38.8–61.4)	99.7 (86.6–112.8)	1333.2 (1186.9–1479.5)
		7–11	427	290.2 (271.6–308.8)	106.2 (84.3–128.1)	4.1 (0.7–7.5)	8.2 (3.5–12.8)	53.7 (13.7–93.7)	58.6 (20.7–96.4)	1154.4 (717.6–1591.2)
		≥ 12	1037	289.4 (277.8–300.9)	139.8 (119.6–160)	15.4 (9.4–21.4)	19.8 (13.4–26.2)	44.7 (24.5–64.8)	63.5 (43.5–83.4)	1373.3 (1123.5–1623.2)
	Occupation	Employed	927	255.1 (243–267.2)	157.2 (131.4–183.1)	11.7 (5.1–18.3)	14.6 (6.8–22.4)	144.5 (104.3–184.7)	207.5 (166.9–248.1)	2767.3 (2318–3216.6)
		Unemployed	4564	295 (288.7–301.2)	105.6 (96.6–114.7)	8.8 (6–11.6)	10.7 (8.1–13.2)	29.6 (21.5–37.6)	63.7 (54.1–73.2)	1026.6 (915.9–1137.4)
	Area	Rural	1336	257.9 (247–268.7)	107.8 (91–124.5)	9 (3.7–14.3)	8.9 (4.2–13.6)	94.2 (66.5–122)	170.9 (142–199.9)	1976 (1652.3–2299.6)
		Urban	4155	297 (290.5–303.5)	116.5 (106.3–126.6)	9.4 (6.4–12.4)	12.1 (9.1–15)	36.1 (26.6–45.5)	64.1 (53.4–74.8)	1134.2 (1009.7–1258.7)
	Insurance	No insurance	289	287.1 (262.4–311.8)	96.3 (60–132.6)	10.1 (−0.5–20.7) ^‡^	13.2 (0.9–25.5)	32.5 (7.8–57.3)	66 (32.3–99.8)	1043.1 (708.7–1377.5)
		Basic	2761	284.5 (276.4–292.7)	108.8 (97.2–120.4)	7.9 (4.5–11.3)	7.7 (4.9–10.6)	61.5 (46.9–76)	110.6 (94.6–126.7)	1463.8 (1289.8–1637.8)
		Complementary	2441	292.1 (283.9–300.3)	122.6 (108.8–136.5)	10.7 (6.5–14.9)	15 (10.7–19.3)	38.1 (24.2–52.1)	67 (51.9–82.1)	1209.3 (1024.2–1394.3)
	Obesity	Normal	2360	275.5 (267.2–283.7)	124.6 (111.2–138.1)	12.1 (7.2–17)	10.5 (6.9–14.1)	66.1 (47.9–84.4)	115.7 (96.4–134.9)	1628.9 (1400.5–1857.4)
		Obese	3131	297.6 (289.9–305.2)	106.9 (95.5–118.3)	7.2 (4.5–9.9)	12 (8.5–15.4)	36.8 (26.8–46.7)	68 (56.2–79.7)	1099.3 (973.5–1225.1)
	Wealth	Q1	1826	288.7 (278.2–299.2)	111.4 (95.4–127.3)	4.7 (1.9–7.4)	7.9 (3.4–12.3)	32.7 (21.6–43.8)	86.8 (70–103.6)	1123.1 (964.3–1281.9)
		Q2	1829	298.9 (288.5–309.3)	106.2 (91.4–120.9)	11.1 (5.4–16.8)	11.4 (6.7–16.1)	60.9 (41.2–80.7)	87.5 (69.2–105.9)	1396.3 (1163.4–1629.2)
		Q3	1836	277.4 (269.1–285.8)	125.1 (110.4–139.7)	11.7 (7.3–16.1)	14.3 (10.5–18.2)	53 (35.6–70.4)	90.5 (70.8–110.1)	1437.7 (1214–1661.4)
	Hypertension	No	2732	272 (264.4–279.5)	127 (114.5–139.5)	11.7 (7.2–16.1)	13.6 (9.8–17.4)	65.5 (49–82)	104.6 (87.9–121.3)	1598.1 (1396.5–1799.8)
		Yes	2759	304.8 (296.5–313.1)	101.6 (89.4–113.7)	6.9 (4.3–9.5)	9 (5.8–12.2)	32.6 (22.9–42.3)	71.6 (58.5–84.7)	1044.5 (912.2–1176.7)
	DM	No	4218	280.4 (274.2–286.5)	122 (111.5–132.5)	9.6 (6.5–12.7)	10.7 (8–13.5)	57.3 (45.2–69.4)	97.7 (85–110.5)	1457.4 (1306.4–1608.4)
		Yes	1273	313.6 (300.7–326.4)	89.8 (75.8–103.8)	8.2 (3.8–12.6)	13.3 (7.6–19)	23.1 (11.7–34.5)	57.6 (39.8–75.3)	893 (737.1–1048.8)
	Cholesterol	No	3730	284.4 (277.7–291.1)	116.4 (105.7–127.1)	9.7 (6.3–13.1)	9.8 (7.1–12.5)	56.5 (43.9–69.1)	97.1 (83.6–110.7)	1423.1 (1265.9–1580.3)
		Yes	1761	296.1 (285.9–306.4)	110.3 (95.4–125.3)	8.4 (4.8–12.1)	14.7 (9.4–20)	33.8 (19.8–47.8)	69.4 (52.9–86)	1115.4 (936–1294.8)
	CVD	No	4340	280.8 (274.7–287)	114.3 (104.8–123.8)	10.8 (7.6–14.1)	11.5 (8.7–14.3)	55.7 (44–67.4)	97.6 (85–110.1)	1425.8 (1281.2–1570.4)
		Yes	1151	315.8 (302.5–329.1)	115.2 (94.1–136.4)	3.4 (1.4–5.5)	10.7 (5–16.5)	25 (12.2–37.8)	53.5 (35.4–71.6)	945.2 (750.2–1140.2)
	Cancer	No	5379	287.4 (281.7–293)	114.2 (105.4–123)	9.3 (6.7–12)	11.5 (8.9–14)	49.7 (39.8–59.5)	89.6 (78.7–100.4)	1333.1 (1209.4–1456.8)
		Yes	112	326.7 (284.2–369.1)	131.9 (62.1–201.7)	6.9 (0–15.1) ^‡^	3.6 (0–7.4) ^‡^	31.3 (3.3–59.3)	26.4 (5.2–47.7)	953.5 (552–1355.1)
	Depression	No	3527	276.4 (269.8–283)	125.6 (114.4–136.8)	9.3 (6.5–12.1)	12.2 (9.2–15.3)	52.7 (41–64.3)	99.9 (85.7–114.1)	1446.6 (1299.8–1593.4)
		Yes	1964	309.5 (299.2–319.8)	94.2 (80.5–107.8)	9.3 (4.1–14.6)	9.7 (5.3–14.1)	43.2 (26.1–60.3)	67.3 (52.1–82.5)	1104.8 (890.2–1319.4)
	Comorbidity^†^	0	1715	262.9 (253.5–272.3)	124 (108.6–139.4)	14.3 (7.5–21.1)	12.1 (7.1–17.1)	79.3 (56–102.6)	119.3 (97.1–141.6)	1770.7 (1488.5–2053)
		1	1665	288.4 (278.6–298.2)	118.7 (102.2–135.2)	7.3 (4.5–10.1)	11.5 (7.7–15.3)	46.7 (30.4–63)	90.8 (71.3–110.4)	1316.2 (1114–1518.5)
		2	1153	296.7 (284.4–309)	111.5 (91.7–131.4)	5 (1.8–8.1)	7 (2.7–11.3)	32.6 (18.2–46.9)	67.8 (47.6–88.1)	1045.8 (838.5–1253.1)
		3+	958	323.8 (308.4–339.1)	93.3 (74.7–112)	8.6 (3.2–14.1)	14.7 (7.3–22)	18.4 (6.1–30.7)	51.4 (32.9–69.9)	853.9 (671–1036.7)
Female	Age (years)	60–69	1851	269 (260.1–278)	83.4 (72.8–94)	6.1 (2.5–9.7)	9 (4.9–13.1)	15.5 (10.1–20.9)	67.2 (53–81.4)	811 (705.6–916.3)
		70–79	697	320.9 (303.8–338)	70 (53.2–86.8)	3 (0–6.1) ^‡^	7 (2.1–12)	10.1 (3.2–16.9)	45.8 (27–64.6)	595.7 (455.4–736)
		≥ 80	217	382.1 (342.9–421.3)	30.7 (11–50.4)	0 (0–0)	0 (0–0)	29.9 (0–66.6) ^‡^	52.3 (9.2–95.5)	571.5 (223.3–919.8)
	Marriage	Married	1694	274.5 (264.8–284.3)	77.4 (66.9–87.8)	5.6 (2.4–8.7)	10.9 (6.1–15.6)	15 (9.4–20.6)	63.9 (50–77.7)	772.9 (674.8–870.9)
		Not married	1071	315.4 (301.5–329.3)	74.1 (59.9–88.2)	3.8 (0–8.2) ^‡^	3 (0.8–5.2)	15.6 (6.5–24.7)	56 (37.3–74.8)	687.6 (537.4–837.9)
	Education (years)	0–6	2316	293.3 (284–302.7)	73.5 (63.9–83.1)	4.2 (1.4–7)	6.3 (3–9.6)	16.9 (10.9–22.8)	62.2 (50.5–73.9)	736.3 (640.7–831.8)
		7–11	138	283.6 (254.7–312.6)	87.3 (54.4–120.3)	0 (0–0)	10.2 (0.6–19.7)	7.1 (0–15.1) ^‡^	12 (3–21.1)	495.2 (332.9–657.5)
		≥ 12	311	276.1 (258.2–293.9)	86.3 (65.4–107.2)	11.2 (1.6–20.9)	15.8 (5.4–26.2)	9.3 (1.5–17.1)	75.1 (30.5–119.7)	873 (638.8–1107.1)
	Occupation	Employed	52	228.2 (190.2–266.3)	109.8 (46–173.5)	1.3 (0–3.8) ^‡^	15.8 (0–43.7) ^‡^	0 (0–0)	115.7 (15.4–216)	975.4 (461.7–1489)
		Unemployed	2713	291.7 (283.5–299.9)	75.4 (66.9–83.9)	4.9 (2.3–7.6)	7.6 (4.6–10.7)	15.6 (10.5–20.6)	59.7 (48.5–70.9)	734.8 (650–819.5)
	Area	Rural	694	272.7 (256.7–288.7)	74.1 (56.5–91.8)	3.7 (0–7.9) ^‡^	6.5 (1.5–11.4)	21.1 (11.9–30.4)	109.1 (81.3–136.9)	957.3 (774.9–1139.6)
		Urban	2071	295.7 (286.3–305)	76.7 (67.1–86.3)	5.2 (2.1–8.3)	8.2 (4.6–11.9)	13.5 (7.7–19.2)	46.4 (34.5–58.2)	674.7 (580.8–768.7)
	Insurance	No insurance	154	295.7 (259.1–332.2)	58.1 (26.5–89.6)	8.7 (0–25.3) ^‡^	14.2 (0–34) ^‡^	30.3 (0.3–60.2)	25.2 (6.3–44.1)	701.6 (321.2–1082)
		Basic	1457	288.6 (277.1–300.2)	76.6 (64.9–88.3)	4.8 (0.7–8.9)	4.5 (2.1–6.9)	15.8 (9.2–22.3)	76.3 (60.5–92.2)	794.1 (670.9–917.4)
		Complementary	1154	291.8 (279.9–303.8)	77.7 (64.6–90.8)	4.5 (1.7–7.3)	10.9 (5.1–16.8)	12.8 (5.5–20.1)	47 (29.6–64.4)	680.7 (563–798.3)
	Obesity	Normal	1097	278 (265.8–290.2)	90.8 (75.6–106.1)	5.6 (1.8–9.4)	6.6 (2.4–10.8)	20.8 (11.4–30.3)	77.2 (55.3–99.1)	910.4 (763.3–1057.4)
		Obese	1668	298.5 (287.7–309.2)	66.5 (56.7–76.3)	4.4 (0.9–7.9)	8.6 (4.4–12.8)	11.6 (6.3–16.9)	50.2 (38.5–61.9)	628.9 (529.6–728.2)
	Wealth	Q1	1035	292.5 (278.2–306.9)	84.8 (67.7–102)	1.1 (0–2.1)	5 (1–8.9)	10.3 (4.5–16.1)	69.6 (51.4–87.7)	728.4 (604.8–852)
		Q2	930	304.6 (289.9–319.3)	64.9 (52.7–77.1)	5.5 (1.1–9.8)	9.2 (2.6–15.9)	19.5 (8.8–30.2)	57.9 (35.9–79.9)	728.1 (583.4–872.9)
		Q3	800	272.7 (260.2–285.1)	78.2 (64.7–91.7)	8.5 (1.8–15.3)	9.5 (4.6–14.4)	16.2 (7.8–24.7)	54.1 (36.8–71.4)	765.3 (597–933.5)
	Hypertension	No	1132	267.2 (255.9–278.6)	81.8 (69.1–94.6)	6.4 (1.6–11.3)	11.1 (6.2–16)	14.8 (7.9–21.8)	71.2 (51.4–91)	827 (685.7–968.3)
		Yes	1633	307.3 (296.1–318.4)	71.9 (60.7–83.1)	3.7 (1–6.5)	5.4 (1.6–9.2)	15.5 (8.7–22.3)	53.3 (40.5–66.1)	676.4 (574.8–777.9)
	DM	No	2004	283.1 (273.9–292.3)	77.4 (67–87.8)	4.4 (1.5–7.3)	6.9 (4–9.8)	16.9 (10.8–22.9)	65.1 (52.3–77.9)	767.7 (666.1–869.3)
		Yes	761	309.2 (292.6–325.8)	72.7 (58.5–86.8)	6 (0.5–11.6)	10.3 (2.4–18.1)	11 (2.9–19.2)	49.8 (27.2–72.4)	667.7 (522.7–812.6)
	Cholesterol	No	1657	290.4 (279.8–300.9)	73.1 (62.4–83.7)	4.1 (0.8–7.4)	4.4 (2.2–6.5)	19.9 (12.3–27.4)	68.8 (52.6–85)	776.4 (657.4–895.3)
		Yes	1108	290.5 (277.8–303.2)	80.7 (67–94.5)	6.1 (1.8–10.3)	13.1 (6.2–20)	8.2 (3.8–12.5)	48.6 (35.2–62.1)	683.6 (575.4–791.8)
	CVD	No	2261	283.7 (275–292.4)	77.9 (68.7–87)	5.6 (2.5–8.7)	7.2 (4.3–10.1)	16.9 (11–22.8)	64.7 (51.9–77.4)	778.6 (681.7–875.5)
		Yes	504	321.2 (300–342.4)	68 (46.5–89.5)	1.6 (0–3.7) ^‡^	10.7 (0.1–21.3)	7.7 (2.9–12.6)	43.3 (21.9–64.7)	561.9 (417.3–706.6)
	Cancer	No	2712	290.3 (282.1–298.5)	76.2 (67.7–84.7)	5 (2.3–7.6)	7.9 (4.8–11)	15.4 (10.4–20.4)	61.4 (50.1–72.8)	744.7 (659.7–829.8)
		Yes	53	295.6 (245.8–345.3)	70.1 (10.7–129.5)	0 (0–0)	4.6 (0–11.6) ^‡^	7.3 (0–19.4) ^‡^	26.8 (0–59.7) ^‡^	464.4 (171.8–756.9)
	Depression	No	1516	279 (268.9–289.2)	86.1 (73.8–98.5)	5 (1.4–8.6)	7.4 (3.9–10.8)	18 (10.7–25.4)	63.5 (49.2–77.7)	811.8 (691.5–932.1)
		Yes	1249	304.8 (291.7–317.9)	63.4 (52.4–74.3)	4.7 (1.1–8.4)	8.4 (3.1–13.6)	11.7 (5.6–17.8)	57.5 (39.7–75.2)	648.5 (535.7–761.3)
	Comorbidity^†^	0	672	267.6 (252.3–282.9)	74.4 (58.4–90.5)	7.5 (0–15.1) ^‡^	6.5 (2.1–10.9)	16.5 (6.3–26.7)	76.3 (51.3–101.3)	820.9 (621.1–1020.6)
		1	843	285.1 (271.1–299.2)	81.9 (65.6–98.2)	3.4 (0.8–6)	8.6 (3.3–13.9)	21.3 (10.3–32.2)	66.8 (44.2–89.3)	826.5 (667.3–985.7)
		2	665	291.9 (275.9–307.9)	73.8 (57.9–89.7)	1.7 (0–3.8) ^‡^	4.5 (0.6–8.3)	15.7 (5.4–26)	55.8 (34.7–77)	675.7 (522.4–829.1)
		3+	585	323.3 (303.2–343.4)	72.1 (52.8–91.5)	7.4 (0.3–14.5)	11.9 (1.8–22)	4.5 (1.3–7.6)	39.5 (22.2–56.8)	589 (450.2–727.9)
Male	Age (years)	60–69	1643	274.3 (264.9–283.8)	155.4 (136.8–174)	15.8 (9.6–22)	16.7 (11.3–22.1)	101.9 (74.4–129.5)	117.2 (94.1–140.3)	2098.9 (1779.4–2418.3)
		70–79	786	297.4 (282.6–312.2)	166.3 (133.5–199.1)	9.5 (3.2–15.7)	12.5 (6.4–18.5)	66.2 (39–93.4)	138.1 (101.3–174.9)	1873 (1498.2–2247.8)
		≥ 80	297	319.7 (290.5–348.9)	106.1 (74.1–138)	14.5 (0–31.5) ^‡^	11.6 (0–26.2) ^‡^	28.7 (0–57.5) ^‡^	50.2 (9.9–90.5)	1016.8 (576.4–1457.2)
	Marriage	Married	2581	283.3 (275.4–291.2)	155.6 (139.8–171.4)	13.9 (9.2–18.7)	14.9 (10.8–19)	86.3 (66.8–105.8)	119.3 (100.6–138.1)	1961.3 (1724.5–2198.1)
		Not married	145	328.2 (287.6–368.8)	117.2 (73.1–161.3)	11.2 (0–25.8) ^‡^	15.4 (0–32.4) ^‡^	42.7 (0–98.9) ^‡^	65.1 (0–135.5) ^‡^	1221.6 (550.8–1892.5)
	Education (years)	0–6	1711	279.4 (269.5–289.4)	155.2 (134.3–176)	13.5 (6.9–20.1)	13.2 (7.8–18.6)	95.8 (70.5–121.2)	151.1 (124.8–177.5)	2152.7 (1837.3–2468.1)
		7–11	289	293.7 (269.7–317.6)	116.2 (87.7–144.7)	6.3 (1.2–11.5)	7.1 (2.1–12.1)	78.4 (17.5–139.2)	83.2 (25.8–140.7)	1503.8 (845.3–2162.2)
		≥ 12	726	295.7 (280.9–310.4)	165.3 (137.5–193.2)	17.4 (9.9–25)	21.7 (13.7–29.7)	61.5 (32.1–90.9)	58 (37.5–78.5)	1611.6 (1262–1961.2)
	Occupation	Employed	875	256.9 (244.2–269.5)	160.3 (133.1–187.5)	12.4 (5.4–19.5)	14.5 (6.4–22.6)	154 (111.3–196.6)	213.6 (170.8–256.3)	2884.5 (2408.5–3360.5)
		Unemployed	1851	299.7 (289.9–309.5)	150.2 (132–168.4)	14.5 (8.7–20.3)	15.1 (10.7–19.6)	50.2 (31.9–68.5)	69.6 (52.7–86.4)	1456.6 (1214.7–1698.5)
	Area	Rural	642	242.4 (228–256.8)	142.8 (114.3–171.2)	14.5 (4.6–24.3)	11.4 (3.3–19.5)	170.3 (115.4–225.2)	235.2 (184.4–286)	3035.6 (2418.9–3652.3)
		Urban	2084	298.3 (289.3–307.4)	156.6 (138.9–174.3)	13.6 (8.5–18.7)	15.9 (11.4–20.5)	58.9 (40.9–76.8)	81.9 (64.1–99.8)	1597.6 (1368.5–1826.7)
	Insurance	No insurance	135	277.7 (244.9–310.5)	138.2 (71.4–204.9)	11.6 (0–24.2) ^‡^	12.2 (0–26) ^‡^	35 (0–75.1) ^‡^	110.7 (44.2–177.2)	1417 (862.3–1971.7)
		Basic	1304	280 (268.5–291.5)	144.3 (123.9–164.7)	11.4 (5.8–17)	11.3 (5.9–16.7)	111.9 (82.5–141.4)	148.5 (119.8–177.3)	2202.9 (1869.4–2536.4)
		Complementary	1287	292.3 (281.1–303.5)	164.1 (140.7–187.5)	16.4 (8.8–24)	18.8 (12.5–25)	61.5 (35.7–87.3)	85.4 (61.3–109.6)	1696.2 (1360.6–2031.8)
	Obesity	Normal	1263	273.3 (262–284.5)	153.9 (132.7–175.2)	17.7 (9.1–26.3)	13.8 (8.1–19.4)	105.3 (72.5–138.2)	149 (118.7–179.4)	2251.3 (1848.8–2653.8)
		Obese	1463	296.5 (285.7–307.3)	153.1 (131.7–174.6)	10.4 (6.4–14.5)	15.9 (10.3–21.5)	65.5 (45.2–85.8)	88.3 (67–109.7)	1637.2 (1396.5–1877.8)
	Wealth	Q1	791	283.6 (268.3–298.9)	146.5 (117.5–175.6)	9.4 (3.2–15.6)	11.7 (2.7–20.7)	62.3 (37.9–86.7)	109.7 (78.9–140.4)	1645.3 (1319.5–1971.1)
		Q2	899	293.1 (278.5–307.7)	148.7 (121.8–175.5)	16.8 (6.2–27.5)	13.6 (7.1–20.2)	103.6 (65.3–141.9)	118.1 (88.8–147.5)	2085.1 (1642.3–2528)
		Q3	1036	281.2 (269.9–292.5)	162.3 (138.6–185.9)	14.3 (8.5–20.1)	18.2 (12.4–24)	82.2 (51.9–112.5)	119.4 (87.1–151.7)	1971.1 (1597.1–2345)
	Hypertension	No	1600	275.4 (265.2–285.5)	159.4 (140.2–178.6)	15.4 (8.6–22.2)	15.4 (9.9–21)	101.8 (74–129.5)	128.5 (103.8–153.3)	2150.9 (1823.4–2478.4)
		Yes	1126	301.2 (288.9–313.4)	144.8 (120.2–169.4)	11.4 (6.5–16.4)	14.2 (8.7–19.7)	57.5 (35.9–79.1)	98.3 (72.3–124.4)	1580.6 (1295.6–1865.6)
	DM	No	2214	277.9 (269.5–286.2)	162.1 (144.7–179.6)	14.3 (9–19.6)	14.2 (9.7–18.8)	93.7 (71.5–115.9)	127.1 (105.8–148.4)	2077.9 (1809.5–2346.2)
		Yes	512	320.2 (299.8–340.6)	115.9 (88–143.8)	11.5 (4.3–18.8)	17.9 (10–25.9)	41.4 (15.6–67.1)	69.4 (40.7–98)	1235.7 (913.3–1558.1)
	Cholesterol	No	2073	279.6 (270.9–288.3)	151.4 (134.3–168.6)	14.2 (8.7–19.8)	14.2 (9.6–18.7)	86.1 (64.3–107.8)	120.1 (99.4–140.7)	1945.2 (1680.7–2209.7)
		Yes	653	305.6 (288.6–322.7)	160.1 (127.9–192.3)	12.4 (5.6–19.1)	17.3 (9.1–25.5)	76.9 (40.5–113.3)	104.4 (66.5–142.4)	1841.7 (1404–2279.4)
	CVD	No	2079	277.7 (268.9–286.4)	154.2 (137.2–171.1)	16.6 (10.7–22.4)	16.2 (11.3–21.1)	98.2 (74.7–121.7)	133.6 (111.4–155.7)	2133.8 (1854.4–2413.1)
		Yes	647	311.7 (294.8–328.6)	151.3 (118.1–184.5)	4.9 (1.6–8.1)	10.8 (4.7–16.9)	38.2 (16.1–60.3)	61.3 (33.9–88.7)	1238.2 (914.8–1561.6)
	Cancer	No	2667	284.4 (276.5–292.2)	152.8 (137.5–168.1)	13.8 (9.2–18.4)	15.2 (11.1–19.3)	84.5 (65.5–103.6)	118.2 (99.7–136.7)	1931.6 (1700.5–2162.7)
		Yes	59	354.2 (289.3–419.1)	186.6 (67.6–305.7)	13.1 (0–28.6) ^‡^	2.6 (0–6.2) ^‡^	52.6 (1.2–104)	26.1 (0–53.6) ^‡^	1386.6 (683.5–2089.7)
	Depression	No	2011	274.4 (265.7–283.1)	156.1 (138.9–173.4)	12.6 (8.5–16.7)	16 (11.3–20.7)	79.4 (59.6–99.2)	128 (105.5–150.6)	1936.7 (1696.6–2176.9)
		Yes	715	317.4 (300.8–334)	146.2 (114.9–177.4)	17.1 (4.4–29.8)	11.9 (4.2–19.7)	96.3 (51.9–140.7)	83.9 (56.2–111.7)	1875.7 (1337.9–2413.5)
	Comorbidity^†^	0	1043	259.9 (248–271.8)	155.8 (133–178.7)	18.6 (8.6–28.7)	15.8 (8.1–23.5)	119.6 (82.2–157)	147 (114.2–179.7)	2380 (1940–2820)
		1	822	291.9 (278.2–305.5)	157.3 (128.5–186.2)	11.4 (6.3–16.5)	14.5 (9.2–19.9)	73.4 (42.1–104.7)	116.1 (84–148.3)	1830.6 (1455.2–2205.9)
		2	488	303.3 (284.1–322.5)	164.2 (122.8–205.6)	9.5 (2.6–16.3)	10.5 (1.7–19.2)	56.1 (25.1–87.1)	84.6 (46.2–123)	1561.5 (1120.6–2002.5)
		3+	373	324.5 (300.7–348.2)	125.7 (89.4–162)	10.5 (2.2–18.8)	18.8 (8.6–29.1)	39.7 (9.1–70.3)	69.5 (31.1–107.9)	1258.1 (851.6–1664.5)

Note: Data are means with 95% confidence intervals (CI) or n. Sedentary behavior is expressed in minutes per day; all other activities (walking for transport, leisure‑time and occupational vigorous/moderate activities) are expressed in minutes per week. Total physical activity is expressed in MET‑adjusted minutes per week.

† Number of comorbid conditions among hypertension, diabetes mellitus, hypercholesterolemia, cardiovascular disease, and cancer.

‡ Because time cannot be less than 0, negative lower CIs were set to 0 in the table.

CI, confidence interval; MET, metabolic equivalent; DM, diabetes mellitus; CVD, cardiovascular disease.

**Table 2:** Prevalence of Physical Inactivity by Participant Characteristics.

Variables		Total Inactive	Female Inactive	Male Inactive
	Subgroups	Prevalence % (95% CI)		
	Total	69.6 (68.3–71.0)		
Sex	Female	76.8 (75.0–78.5)		
	Male	62.4 (60.3–64.4)		
Age (years)	60–69	67.8 (66.0–69.5)	74.1 (71.9–76.3)	60.5 (57.8–63.1)
	70–79	70.3 (67.7–73.0)	80.6 (77.3–83.8)	61.6 (57.9–65.4)
	≥ 80	80.9 (77.1–84.7)	88.2 (83.3–93.1)	75.4 (69.9–80.9)
Marriage	Married	67.5 (65.9–69.1)	75.9 (73.6–78.2)	62.0 (59.9–64.1)
	Not married	77.0 (74.4–79.6)	78.2 (75.5–80.9)	68.3 (59.8–76.7)
Education (years)	0–6	72.0 (70.4–73.5)	78.2 (76.3–80.0)	63.5 (60.9–66.1)
	7–11	71.1 (66.5–75.7)	78.0 (70.7–85.3)	67.5 (61.6–73.3)
	≥ 12	61.2 (57.9–64.5)	68.1 (62.5–73.7)	58.0 (53.9–62.0)
Occupation	Employed	57.9 (54.4–61.4)	58.5 (43.6–73.3)	57.9 (54.2–61.5)
	Unemployed	72.1 (70.6–73.5)	77.2 (75.4–78.9)	64.5 (62.1–67.0)
Area	Rural	66.8 (63.9–69.6)	73.2 (69.5–76.9)	60.1 (55.8–64.4)
	Urban	70.5 (68.9–72.0)	77.9 (75.9–79.9)	63.0 (60.7–65.3)
Insurance	No insurance	74.2 (68.7–79.8)	81.0 (74.3–87.7)	66.8 (58.0–75.6)
	Basic	69.0 (67.1–70.9)	75.5 (73.1–78.0)	61.9 (58.9–64.8)
	Complementary	69.8 (67.7–71.8)	77.8 (75.1–80.4)	62.4 (59.4–65.4)
Obesity	Normal	67.8 (65.7–69.9)	74.5 (71.6–77.3)	62.0 (59.0–65.0)
	Obese	71.0 (69.2–72.8)	78.3 (76.1–80.5)	62.7 (59.9–65.5)
Wealth	Q1	72.1 (69.8–74.4)	77.2 (74.4–80.1)	65.3 (61.6–69.1)
	Q2	70.2 (67.9–72.6)	77.0 (74.0–80.0)	63.3 (59.7–66.8)
	Q3	66.9 (64.5–69.3)	76.1 (72.8–79.3)	59.6 (56.3–63.0)
Hypertension	No	66.7 (64.7–68.6)	74.1 (71.3–76.9)	61.3 (58.6–64.0)
	Yes	72.7 (70.8–74.6)	78.7 (76.5–80.9)	64.0 (60.8–67.1)
DM	No	68.1 (66.6–69.7)	76.4 (74.4–78.5)	60.7 (58.4–63.0)
	Yes	74.5 (71.8–77.2)	77.7 (74.4–81.1)	69.5 (65.1–74.0)
Cholesterol	No	69.3 (67.6–70.9)	77.3 (75.1–79.5)	62.8 (60.5–65.1)
	Yes	70.4 (67.9–72.8)	76.0 (73.1–78.8)	61.0 (56.6–65.3)
CVD	No	68.5 (67.0–70.1)	75.9 (74.0–77.9)	60.4 (58.0–62.8)
	Yes	73.9 (71.1–76.7)	80.8 (76.9–84.6)	68.7 (64.7–72.7)
Cancer	No	69.6 (68.2–71.0)	76.8 (75.0–78.5)	62.3 (60.2–64.3)
	Yes	72.4 (63.1–81.6)	78.6 (66.3–91.0)	66.8 (53.4–80.3)
Depression	No	67.1 (65.4–68.8)	74.5 (72.0–76.9)	61.4 (59.0–63.8)
	Yes	74.2 (72.1–76.4)	79.7 (77.2–82.2)	65.0 (61.1–68.9)
Comorbidity^*†*^	0	66.1 (63.6–68.6)	75.7 (72.1–79.2)	60.0 (56.7–63.3)
	1	68.4 (65.9–70.9)	74.9 (71.6–78.1)	61.6 (57.8–65.4)
	2	73.6 (70.8–76.4)	79.6 (76.2–83.0)	65.2 (60.4–70.0)
	3+	73.6 (70.4–76.7)	77.7 (73.9–81.5)	67.3 (62.0–72.6)

Note: Data presents the prevalence of physical inactivity with 95% confidence intervals (CI). defined as accumulating fewer than 600 MET × min per week of physical activity.

† Number of comorbid conditions among hypertension, diabetes mellitus, hypercholesterolemia, cardiovascular disease, and cancer.

CI, confidence interval; MET, metabolic equivalent; DM, diabetes mellitus; CVD, cardiovascular disease.

**Fig. 2:**
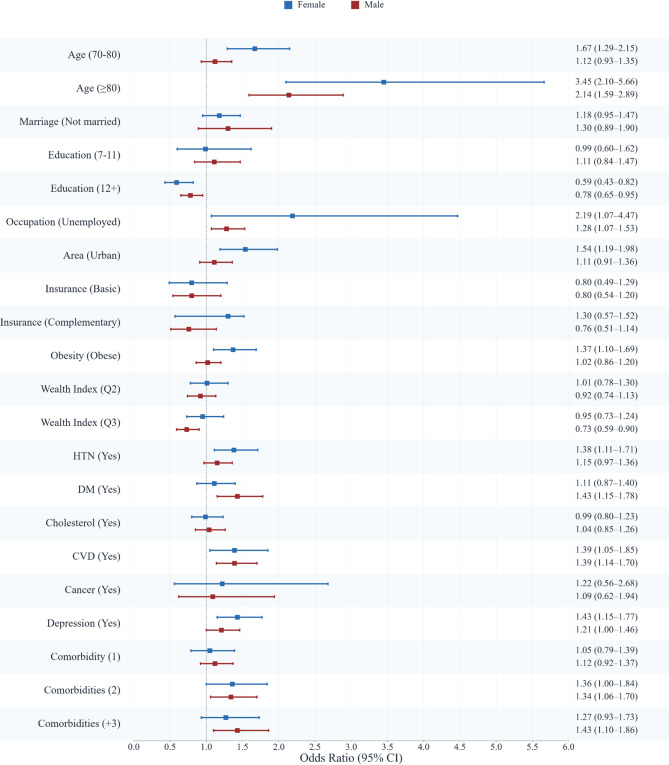
Forest Plot Presenting Unadjusted OR and 95% CI Note: The plot displays the unadjusted odds ratios (OR) and their corresponding 95% confidence intervals (CI) for the association between various sociodemographic, lifestyle, and health factors and the likelihood of being physically inactive. The analysis is stratified by sex, with females represented in blue and males in red. The vertical line at an OR of 1.0 indicates no effect. Data points to the right of this line suggest an increased odd of physical inactivity, while points to the left suggest a decreased odd. HTN, hypertension; DM, diabetes mellitus; CVD, cardiovascular disease.

Given the substantial influence of sex, regression analyses focused on sex-stratified generalized linear mixed-effects models with random effects (Fig. 2). In these models, men exhibited lower odds of inactivity than women (unadjusted OR 0.45, 95% CI 0.40–0.52; adjusted OR 0.48, 95% CI 0.42–0.56).

Older age groups had higher odds of inactivity. Compared with adults aged 60–69 years, those aged ≥ 80 years had higher odds in the total sample (adjusted OR 2.47, 95% CI 1.89–3.23), with a stronger effect among women (adjusted OR 3.20, 95% CI 1.96–5.24) than men (adjusted OR 1.91, 95% CI 1.41–2.60).

Higher education (≥ 12 years) was associated with lower odds of inactivity (adjusted OR 0.73, 95% CI 0.61–0.88), with a more pronounced effect in women (adjusted OR 0.63, 95% CI 0.45–0.88). Urban residence had higher odds of inactivity (adjusted OR 1.35, 95% CI 1.13–1.61), particularly among women (adjusted OR 1.62, 95% CI 1.25–2.11). Unemployment was associated with higher odds of inactivity in men (adjusted OR 1.20, 95% CI 1.00–1.44). Wealth effects differed by sex. The third wealth tertile was associated with higher odds of inactivity in women (adjusted OR 1.41, 95% CI 1.14–1.75) but lower odds in men (adjusted OR 0.76, 95% CI 0.61–0.94).

Clinical and psychosocial factors were also associated with inactivity. Obesity was related to higher odds in females (adjusted OR 1.34, 95% CI 1.08–1.66). Depressive symptoms were also associated with inactivity (unadjusted OR 1.54, 95% CI 1.33–1.77; adjusted OR 1.30, 95% CI 1.12–1.51), with a stronger association in women (adjusted OR 1.41, 95% CI 1.14–1.75). A graded association was observed for multimorbidity. Compared with no chronic conditions, having two comorbidities (adjusted OR 1.29, 95% CI 1.06–1.56) and three or more (adjusted OR 1.26, 95% CI 1.02–1.55) was associated with higher odds. Full model coefficients and confidence intervals are reported in Tables 3 and 4.

**Table 3:** Unadjusted regression analysis of factors associated with physical inactivity.

Variables		Total	Female	Male
	Subgroups	OR (95% CI)		
Sex	Male	0.45 (0.40–0.52)		
Age (years)	70–79	1.23 (1.06–1.43)	1.67 (1.29–2.15)	1.12 (0.93–1.35)
	≥ 80	2.21 (1.71–2.85)	3.45 (2.10–5.66)	2.14 (1.59–2.89)
Marriage	Not married	1.73 (1.46–2.05)	1.18 (0.95–1.47)	1.30 (0.89–1.90)
Education (years)	7–11	0.86 (0.67–1.10)	0.99 (0.60–1.62)	1.11 (0.84–1.47)
	≥ 12	0.57 (0.48–0.67)	0.59 (0.43–0.82)	0.78 (0.65–0.95)
Occupation	Unemployed	1.92 (1.62–2.28)	2.19 (1.07–4.47)	1.28 (1.07–1.53)
Area	Urban	1.24 (1.05–1.47)	1.54 (1.19–1.98)	1.11 (0.91–1.36)
Insurance	Basic	0.82 (0.60–1.13)	0.80 (0.49–1.29)	0.80 (0.54–1.20)
	Complementary	0.80 (0.58–1.10)	1.30 (0.57–1.52)	0.76 (0.51–1.14)
Obesity	Obese	1.23 (1.08–1.41)	1.37 (1.10–1.69)	1.02 (0.86–1.20)
Wealth index	Q2	0.92 (0.78–1.09)	1.01 (0.78–1.30)	0.92 (0.74–1.13)
	Q3	0.73 (0.62–0.86)	0.95 (0.73–1.24)	0.73 (0.59–0.90)
Hypertension	Yes	1.43 (1.25–1.63)	1.38 (1.11–1.71)	1.15 (0.97–1.36)
DM	Yes	1.39 (1.19–1.63)	1.11 (0.87–1.40)	1.43 (1.15–1.78)
Cholesterol	Yes	1.18 (1.02–1.35)	0.99 (0.80–1.23)	1.04 (0.85–1.26)
CVD	Yes	1.29 (1.10–1.52)	1.39 (1.05–1.85)	1.39 (1.14–1.70)
Cancer	Yes	1.08 (0.68–1.73)	1.22 (0.56–2.68)	1.09 (0.62–1.94)
Depression	Yes	1.54 (1.33–1.77)	1.43 (1.15–1.77)	1.21 (1.00–1.46)
Comorbidity^†^	1	1.21 (1.03–1.42)	1.05 (0.79–1.39)	1.12 (0.92–1.37)
	2	1.58 (1.31–1.90)	1.36 (1.00–1.84)	1.34 (1.06–1.70)
	3+	1.60 (1.31–1.95)	1.27 (0.93–1.73)	1.43 (1.10–1.86)

Note: This table shows the results of the univariable logistic regression analysis. It presents the unadjusted odds ratios (OR) with their corresponding 95% confidence intervals (CI).

† Number of comorbid conditions among hypertension, diabetes mellitus, hypercholesterolemia, cardiovascular disease, and cancer. CI, confidence interval; OR, odds ratio; DM, diabetes mellitus; CVD, cardiovascular disease.

**Table 4:** Adjusted regression analysis of factors associated with physical inactivity.

Variables		Total	Female	Male
	Subgroups	OR (95% CI)		
Sex	Male	0.48 (0.42–0.56)	-	-
Age	70–79	1.29 (1.10–1.51)	1.63 (1.26–2.11)	1.05 (0.87–1.27)
	≥ 80	2.47 (1.89–3.23)	3.20 (1.96–5.24)	1.91 (1.41–2.60)
Education	7–11	1.09 (0.84–1.41)	1.07 (0.65–1.77)	-
	≥ 12	0.73 (0.61–0.88)	0.63 (0.45–0.88)	-
Occupation	Unemployed	-	-	1.20 (1.00–1.44)
Area	Urban	1.35 (1.13–1.61)	1.62 (1.25–2.11)	-
Obesity	Obese	-	1.34 (1.08–1.66)	-
Wealth index	Q2	-	-	0.93 (0.75–1.16)
	Q3	-	-	0.76 (0.61–0.94)
Depression	Yes	1.30 (1.12–1.51)	1.41 (1.14–1.75)	-
Comorbidity^†^	1	1.06 (0.89–1.25)	-	1.10 (0.90–1.35)
	2	1.29 (1.06–1.56)	-	1.30 (1.02–1.66)
	3+	1.26 (1.02–1.55)	-	1.41 (1.08–1.85)

This table presents the results from the multivariable generalized linear mixed-effects model. It shows the adjusted odds ratios (OR) and their 95% confidence intervals (CI) after adjusting for covariates.

† Number of comorbid conditions among hypertension, diabetes mellitus, hypercholesterolemia, cardiovascular disease, and cancer. CI, confidence interval; OR, odds ratio.

**Fig. 3:**
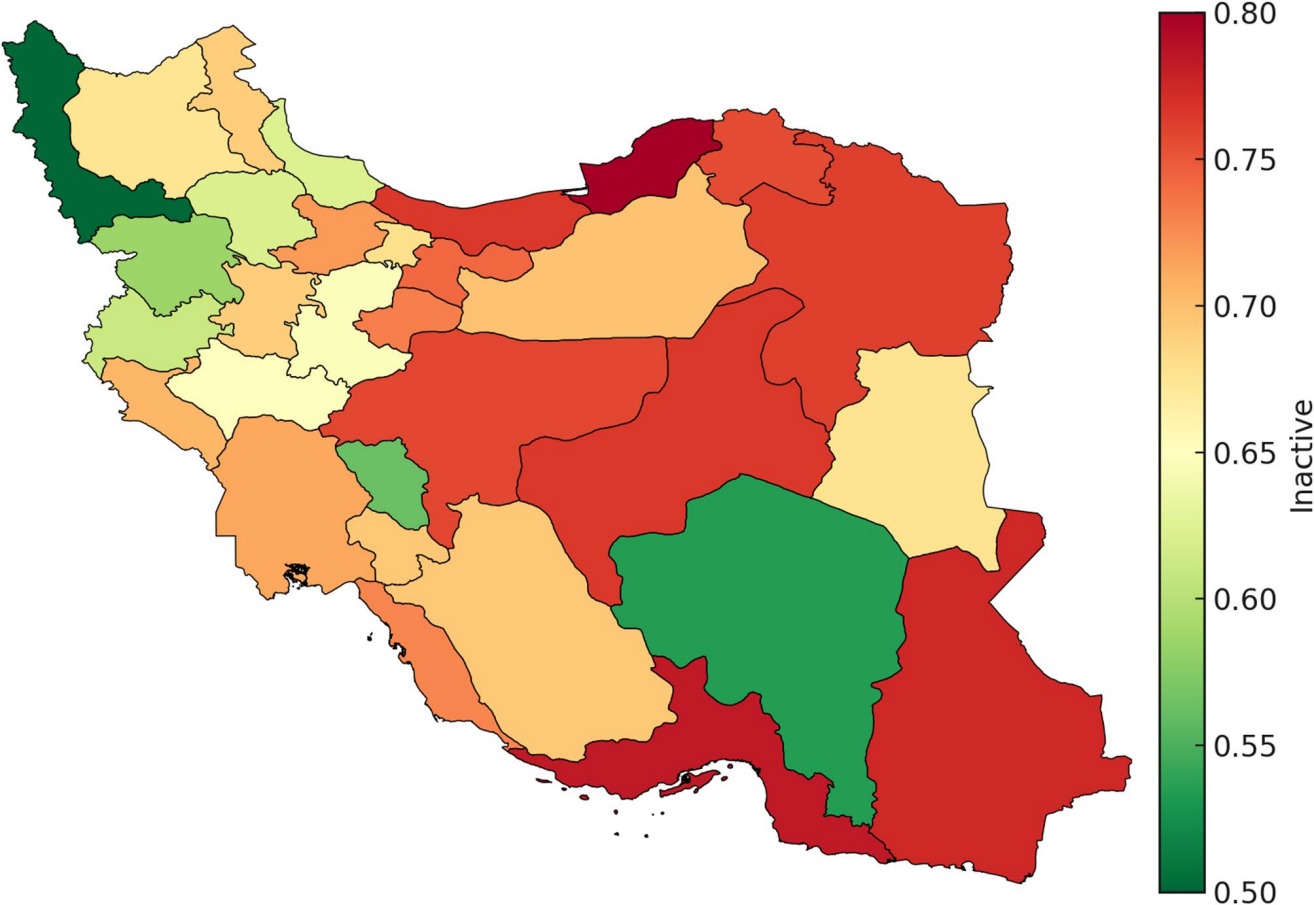
Provincial Prevalence of Physical Inactivity Among Iranian Adults Aged ≥60 Years (STEPS 2021) Note: Choropleth shows the proportion inactive by province (green, lower; red, higher).

Provincial prevalence ranged widely (≈ 50%–81%). Highest levels were observed in Golestan (~ 0.81), Hormozgan (~ 0.78), and Sistan & Baluchistan (~ 0.78), while the lowest were in West Azerbaijan (~ 0.50), Kerman (~ 0.54), and Chaharmahal & Bakhtiari (~ 0.56). See Fig. 3 and supplementary tables for further details. 

**Fig. 4:**
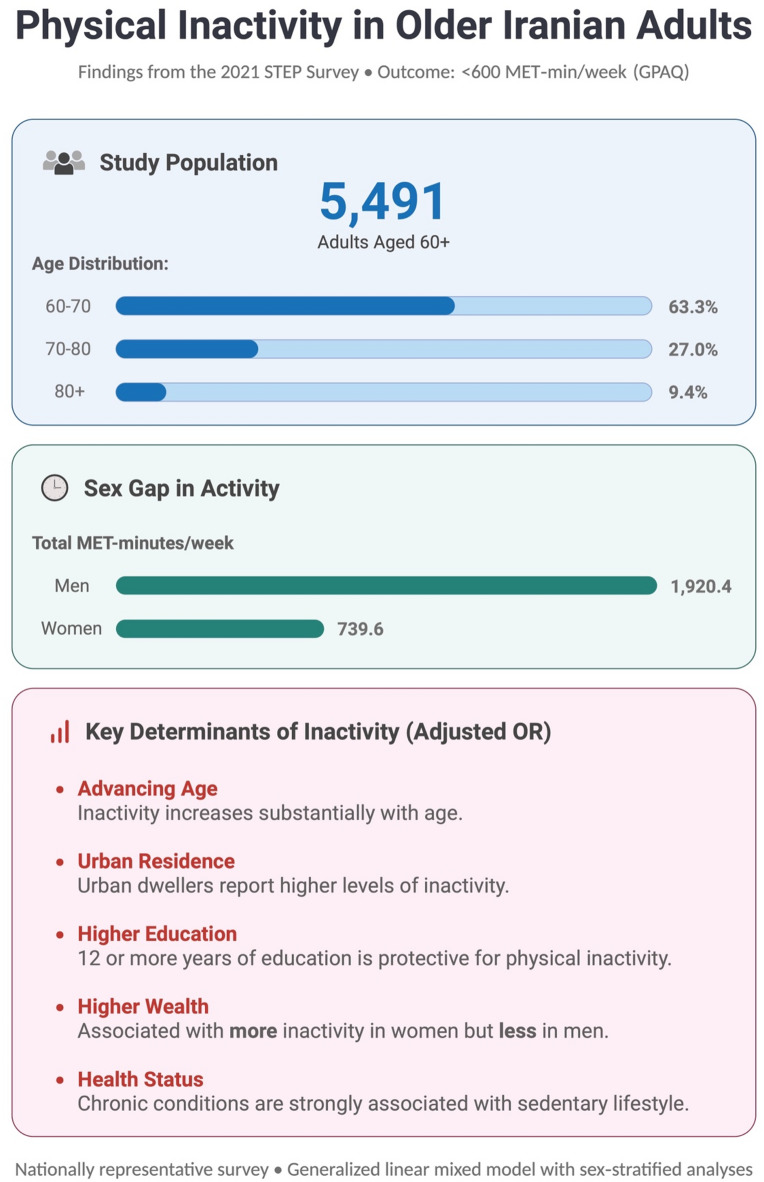
Graphical Overview of the Study Design and Key Findings Abbreviations: GPAQ, global physical activity questionnaire; OR, odds ratio.

Figure 4 provides a graphical overview of the study design and key findings.

## Discussion

Physical inactivity among older adults is a critical public health issue, contributing markedly to the burden of NCDs, functional decline, and reduced quality of life^5^. Our study reveals a high prevalence of physical inactivity among adults aged 60 and over. These estimates are markedly above the global average of 43.5% insufficient activity reported in 2022 and also higher than contemporary figures for many high-income settings^25^. Several factors may help explain the observed differences. Ambient weather conditions may be related to physical activity levels. Mean mid-summer temperatures in Iran approximate 30 °C^26^, and evidence from device-based measurements and systematic reviews consistently reveals an inverse relationship between temperature and physical activity levels^27,28^. Contextual features of the built environment and transportation systems may also be linked to activity patterns. Findings from observational studies indicate that urban designs promoting walkability, are associated with higher levels of moderate-to-vigorous physical activity (MVPA)^29,30^. Air quality is another critical factor. Objective investigations in older adult populations demonstrate that higher particulate matter 2.5 (PM2.5) concentrations and air quality index (AQI) values are associated with fewer daily steps and diminished MVPA^31^. This is highly relevant in Iran’s urban centers; where in 2021, for instance, clean air days accounted for just 1.1% of the year in Tehran. Beyond the national average, we observed pronounced provincial heterogeneity. This heterogeneity suggests local context—environment, climate, urban form, and service availability—may shape older adults’ activity patterns and should inform provincial targeting.

A striking finding from our study was the significant sex disparity, with women being considerably more likely to be physically inactive and reporting more sedentary time than men. This is consistent with global trends, where a higher prevalence of insufficient physical activity is consistently reported among women compared to men^25^. Research has shown that older men often record higher daily step counts and more MVPA^32^. However, it is worth noting that some studies using objective measures like accelerometers have found women to be more active, possibly by capturing more light-intensity household activities that are often underreported in questionnaire-based studies like ours^33^. Several factors may help explain this se gap. In our study, men engaged in significantly more work-related physical activity, which aligns with traditional gender roles^34^. Societal and cultural norms can further shape activity choices, with men often preferring competitive or vigorous activities while women favor social activities closer to home^35^. Furthermore, women face distinct barriers, including a greater fear of injury or falling, which has been highlighted as a significant deterrent to activity^36^. Socioeconomic factors also compound these differences; in our study, urban residence and obesity showed stronger association of inactivity for women, and other research has found that women are more likely to report low income and living alone, which can limit access to physical activity opportunities^37^. Given these pronounced disparities, there is an urgent need for gender-specific interventions that address the barriers, such as supervised group activities that promote safety and social support.

In our data, physical inactivity was more common in older age groups. The odds of being inactive were substantially higher in the oldest age groups compared to those in their sixties, a finding that mirrors previous reports of age-related declines in activity^3,25^. This age trend was markedly more pronounced in women, who already face a higher baseline risk of inactivity. Several factors may help explain these patterns. Deteriorating mobility, driven by age-related loss of muscle strength, joint stiffness, and chronic pain, directly limits the capacity for exercise. This is often compounded by a higher burden of comorbidities in the oldest age groups, which not only reduces physical capacity but also fosters sedentary behavior^38^. Social factors, such as the loss of social networks and support systems, can further reduce motivation and opportunities for activity^39^. Therefore, targeted public health strategies are crucial. Community-based programs that focus on low-impact, mobility-supporting exercises like walking, swimming, or strength training may be appropriate targets for promoting physical activity^40,41^.

We also identified significant urban-rural disparities, with older adults in urban areas exhibiting a higher likelihood of inactivity compared to their rural counterparts. This effect was again more pronounced among women. These findings align with research that points to environmental and lifestyle factors as key determinants of activity levels, although the literature is not entirely consistent, suggesting context-specific influences. While our study found urban residents to be less active, potentially due to their lower engagement in work-related physical activity, other studies have reported the opposite^42^. Urban environments can present unique barriers, such as limited access to green spaces, high traffic density, and perceived safety risks, which may deter outdoor activity^29^. Conversely, while rural areas may lack structured facilities, natural environments or lifestyle factors, such as agricultural work, may facilitate higher overall activity levels^43^.

The influence of socioeconomic factors on physical inactivity was complex and notably sex-specific. Our results showed that higher educational attainment had a protective effect against inactivity, a benefit that was even more pronounced in women. In contrast, the relationship with wealth was divergent, higher wealth was associated with increased odds of inactivity in women but decreased odds in men. This underscores that public health interventions must be tailored by sex, moving beyond simple demographic stratification to explore the underlying mechanisms driving these differences. Education is a fundamental determinant of health, fostering health literacy, self-efficacy, and problem-solving skills that empower individuals to adopt and maintain healthy behaviors^44^. For women in particular, education may equip them with superior psychological tools and strategies to navigate barriers like caregiving responsibilities and time constraints, which disproportionately limit their exercise opportunities.

Counterintuitive findings on wealth reveals how socioeconomic status interacts differently with domains of physical activity across genders. For men, higher wealth may correspond to a shift from manual labor to sedentary professional roles, reducing occupational activity but freeing up time and resources for leisure-time physical activity, such as gym memberships or sports, resulting in a net protective effect^45^. Conversely, wealthier women may substitute physically demanding household or occupational work with domestic help and private transport without a corresponding increase in leisure-time physical activity. Persistent cultural norms and caregiving responsibilities may limit their ability to convert financial resources into more exercise, paradoxically increasing their risk of inactivity^46^.

We observed progressively higher odds of inactivity with increasing comorbidity burden. Hypertension and DM showed particularly strong relationships with inactivity. This observation is consistent with longitudinal research showing that a greater multimorbidity burden predicts progressively lower physical activity trajectories over time^47^. Other analyses have confirmed that adults with multiple chronic conditions, especially cardiometabolic clusters, are significantly more likely to fall below recommended activity guidelines^48^. The barriers are multifaceted, including fear of symptom exacerbation (such as exercise-induced hypertensive spikes or hypoglycemia), low self-efficacy, fatigue, and limited health literacy. Addressing these complex obstacles requires tailored interventions that combine patient education, graded exercise prescriptions, and close clinical monitoring to safely promote sustained physical activity among individuals with a high burden of chronic disease.

The evidence strongly suggests that tailored, multi-component interventions can successfully increase physical activity among older adults. The most effective strategies often combine education, motivational support, and environmental changes^49,50^. Our study provides several key insights for health systems facing rapid aging and urbanization. The pronounced sex disparity in inactivity requires gender-responsive strategies, such as women-only sessions and female instructors and peer mentors. Integrating activity counseling into primary care can effectively target high-risk older adults, particularly those with multimorbidity, obesity, or depression. Furthermore, higher urban inactivity highlights the need for city-level policies like walkable neighborhoods and active transport, while significant subnational heterogeneity demands local surveillance to tailor interventions. Finally, contextual factors like heat or air pollution necessitate flexible alternatives, such as indoor or timed activities.

### Strengths and limitations

The strengths of this study include the use of a nationally representative sample and standardized WHO STEPS procedures, which support comparability with other population surveillance studies. In addition, the GPAQ is a widely used surveillance instrument with evidence of acceptable reliability and validity across multiple settings. We also conducted sex-stratified analyses to provide sex-specific estimates and reduce residual confounding by sex.

However, several limitations should be considered. First, the cross-sectional design precludes causal inference. Second, physical activity and sedentary behavior were assessed using the self-reported GPAQ, which is subject to recall and social desirability bias and may lead to misclassification of activity levels. Systematic evidence shows that self-report measures can differ substantially from device-based measures, often overestimating physical activity compared with accelerometry^51^. Consistent with this concern, a validation study comparing GPAQ with accelerometer data reported notable disagreement for sedentary behavior and evidence of reporting bias, including differential error by activity level^52^. Third, GPAQ primarily captures moderate- and vigorous-intensity activities across work and leisure domains, plus transport-related activity and sitting time, and therefore may under-capture low-intensity and sporadic activities (e.g., light household tasks), which are particularly relevant in older adults^53^. Future studies could strengthen measurement by combining GPAQ with objective monitoring (e.g., accelerometry) to better characterize light-intensity activity patterns and sedentary time.

## Conclusion

Our study highlights the alarmingly high prevalence of physical inactivity among older adults in Iran, particularly among women, the oldest age groups, urban residents, and those with obesity, depression, or multiple comorbidities. These findings underscore the urgent need for targeted public health interventions to promote physical activity in this vulnerable population. Future research should employ longitudinal designs to establish causality and evaluate the effectiveness of interventions, particularly those tailored to high-risk groups. Such efforts are critical for global active aging.

## Supplementary Information

Below is the link to the electronic supplementary material.

Supplementary Material 1

## Data Availability

Further data is available upon reasonable request due to privacy/ethical restrictions.

## Declarations

### AI-assisted technologies statement

The authors used a generative AI tool to improve language and readability. The authors reviewed and edited the content as needed and take full responsibility for the integrity of the work.
