# Supplementary material for: Physical inactivity prevalence and associated factors among iranian older adults in the 2021 STEPS survey
Source: Sci Rep. 2026 Mar 6;16:12296. doi: 10.1038/s41598-026-42828-x (PMC13079872; doi:10.1038/s41598-026-42828-x)
Supplement: Supplementary file 1 — Supplementary Material 1 [file 41598_2026_42828_MOESM1_ESM.docx]

# Province Activity Table

| code | province | inactive | active |
| --- | --- | --- | --- |
| 0 | Markazi | 0.647 | 0.353 |
| 1 | Gilan | 0.624 | 0.376 |
| 2 | Mazandaran | 0.765 | 0.235 |
| 3 | Azerbaijan, East | 0.675 | 0.325 |
| 4 | Azerbaijan, West | 0.499 | 0.501 |
| 5 | Kermanshah | 0.612 | 0.388 |
| 6 | Khuzestan | 0.713 | 0.287 |
| 7 | Fars | 0.694 | 0.306 |
| 8 | Kerman | 0.535 | 0.465 |
| 9 | Khorasan, Razavi | 0.762 | 0.238 |
| 10 | Isfahan | 0.758 | 0.242 |
| 11 | Sistan and Baluchistan | 0.775 | 0.225 |
| 12 | Kurdistan | 0.586 | 0.414 |
| 13 | Hamadan | 0.69 | 0.31 |
| 14 | Chahar Mahaal and Bakhtiari | 0.563 | 0.437 |
| 15 | Lorestan | 0.652 | 0.348 |
| 16 | Ilam | 0.704 | 0.296 |
| 17 | Kohgiluyeh and Boyer-Ahmad | 0.694 | 0.306 |
| 18 | Bushehr | 0.728 | 0.272 |
| 19 | Zanjan | 0.622 | 0.378 |
| 20 | Semnan | 0.698 | 0.302 |
| 21 | Yazd | 0.766 | 0.234 |
| 22 | Hormozgan | 0.783 | 0.217 |
| 23 | Tehran | 0.742 | 0.258 |
| 24 | Ardabil | 0.69 | 0.31 |
| 25 | Qom | 0.731 | 0.269 |
| 26 | Qazvin | 0.718 | 0.282 |
| 27 | Golestan | 0.81 | 0.19 |
| 28 | Khorasan, North | 0.756 | 0.244 |
| 29 | Khorasan, South | 0.676 | 0.324 |
| 30 | Alborz | 0.679 | 0.321 |
